# Implicit Age Cues in Resumes: Subtle Effects on Hiring Discrimination

**DOI:** 10.3389/fpsyg.2017.01321

**Published:** 2017-08-10

**Authors:** Eva Derous, Jeroen Decoster

**Affiliations:** ^1^Department of Personnel Management, Work, and Organizational Psychology, Faculty of Psychology and Educational Sciences, Ghent University Ghent, Belgium; ^2^Thomas More University College Antwerp, Belgium

**Keywords:** age, anonymous resume screening, hiring discrimination, job market signaling theory, recruitment

## Abstract

Anonymous resume screening, as assumed, does not dissuade age discriminatory effects. Building on job market signaling theory, this study investigated whether older applicants may benefit from concealing explicitly mentioned age signals on their resumes (date of birth) or whether more implicit/subtle age cues on resumes (older-sounding names/old-fashioned extracurricular activities) may lower older applicants’ hirability ratings. An experimental study among 610 HR professionals using a mixed factorial design showed hiring discrimination of older applicants based on implicit age cues in resumes. This effect was more pronounced for older raters. Concealing one’s date of birth led to overall lower ratings. Study findings add to the limited knowledge on the effects of implicit age cues on hiring discrimination in resume screening and the usefulness of anonymous resume screening in the context of age. Implications for research and practice are discussed.

## Introduction

In Western society people need to work long enough to maintain welfare levels (Administration on Aging, 2015). Many people also prefer to stay active in the labor market until an older age (Wöhrmann et al., 2016). However, and despite anti-discrimination legislation, chronologically older compared to younger job applicants still have lower chances to hold and obtain jobs, even when their competencies are alike (Neumark et al., 2016; Wanberg et al., 2016).

The present paper focuses on age discrimination in hiring, and more in particular on resume screening, (Bal et al., 2011; Truxillo et al., 2015). Worldwide, resumes are one of the most frequently used screening tools that encompass the first selection hurdle. Moreover, due to the way impressions are formed, this hurdle seems vulnerable for hiring discrimination (Fiske et al., 2002). Although chronological age has no validity for predicting future job performance (Schmidt et al., 2016), correspondence audit studies^1^ consistently show that explicitly presenting one’s chronological age in a resume may decline older applicants’ job chances (Riach and Rich, 2006, 2010; Richardson et al., 2013; Neumark et al., 2016). Moreover, such ageism effects seem substantial. Ahmed et al. (2012), for instance, found that younger (31 years) compared to equally qualified older (46 years) applicants received over three times more responses from employers looking to hire restaurant workers and over four times more responses from employers looking for sales assistants.

Anti-discrimination regulations have not prevented bias in resume screening; therefore AAP are offered to combat illegal discriminatory hiring practices (Åslund and Skans, 2012). AAP like blind auditions (Goldin and Rouse, 2000), blind interviewing (Buijsrogge et al., 2016), and anonymous resume screening (Åslund and Skans, 2012), aim to blot non-job-related, personal identifiers (like socio-demographic information) to increase protected job applicants’ chances of advancing to the next assessment stage, and hence, to increase their hiring chances. Intriguingly, however, results of AAP are mixed. Anonymous resume screening also *dilutes* hiring chances of job applicants from protected social groups (Krause et al., 2012; Behaghel et al., 2015), which suggests that resumes in which demographic information is blotted still reveal information about job applicants’ group membership, albeit in subtle ways. To the best of our knowledge, the role of implicit markers of applicants’ social group membership on their hiring chances has not been investigated much in recruitment (i.e., resume screening), especially not regarding applicants’ older age, and will be considered here.

According to the signaling theory (like the job market signaling theory; see Spence, 1973, 1974; Rynes, 1991) senders (like applicants) exchange information with receivers (like recruiters) through signals/cues (like resume information), which correlate with unobservable characteristics of the sender (Connelly et al., 2011; Bangerter et al., 2012). Hence building on assumptions from both job market signaling theory and impression formation theory, we first investigate whether older job applicants benefit from concealing explicitly mentioned age cues in their resumes (like date of birth) or whether more implicit/subtle age cues in resumes – i.e., other than one’s date of birth- may lower older job applicants’ hirability ratings. Second, surprisingly and with a few exceptions (see Fasbender and Wang, 2017), few studies investigated recruiter characteristics on hiring discrimination, and ageism in particular. Yet, recruiters might differ in their susceptibility to hiring bias and recruiters’ chronological age has been suggested as one potential boundary condition. As a second aim, we therefore explore the potential moderating role of recruiters’ chronological age on implicit/subtle age-related information in resumes on the one hand and applicants’ hirability ratings on the other hand. Some studies showed evidence for in-group favoritism with chronologically older recruiters favoring older applicants (Krings et al., 2011), whereas others have shown the opposite (Finkelstein and Burke, 1998; Axt et al., 2014). Since hiring literature is inconclusive and mainly considered explicit age cues, we further explore whether older recruiters may (dis)favor resumes based on implicit old-age cues (Axt et al., 2014; Marcus and Sabuncu, 2016).

In the next paragraphs, we first discuss why resume screening is vulnerable to age discrimination, what is known about ageism effects in this stage of the hiring procedure, and why anonymous resume screening may fail to avert age discrimination. Next, we turn our attention to job market signaling theory and discuss ‘implicit’ age cues in resumes (i.e., applicants’ name and affiliations). Finally, we explore the potential moderating role of recruiters’ chronological age on implicit age cues and hiring discrimination.

## Theoretical Background

### Age Discrimination in Resume Screening

Resumes are one of the first and most important sources of information when HR managers and recruiters initially screen applicants for jobs, but they also appear very vulnerable to bias (Derous et al., 2015). Job applicants are typically judged on the basis of a one- or two-page resume. This resume screening provides limited individuating information and is vulnerable to categorization effects. That is, cognitive models of impression formation (like the continuum model; see Fiske et al., 1999) suggest that category-based information processing will be particularly strong when limited individualized information is available, such as on resumes (Abrams et al., 2016). Models of impression formation further suggest that categorization will occur automatically and once people have categorized someone as belonging to a particular out-group, associated group stereotypes may be activated, which can influence how people judge others (Fiske et al., 1999).

In many Western European societies, it is common to indicate date of birth on resumes, which explicitly signals applicants’ chronological age. Such an explicit signal might provide recruiters with information about one’s life/work experiences but at the same time might come with a cost and lower older applicants’ hiring chances. When explicit age markers are present in resumes, recruiters seem to prefer younger applicants over older ones as evidenced by many recently conducted (field) experiments (Lahey, 2008; Albert et al., 2011; Krings et al., 2011; Ahmed et al., 2012; Richardson et al., 2013). For instance, Lahey (2008) showed in a field experiment (i.e., correspondence audit study) that chronologically older women received fewer positive reactions to their applications than comparable younger women. In another correspondence audit study, Albert et al. (2011) sent out resumes of equally qualified 24-, 28-, and 38-years old applicants in response to existing job ads. Resumes from 38-years old applicants received a significant lower callback than those from the former two age groups. Similarly, Ahmed et al. (2012) found 46-years old applicants to get lower callback than applicants aged 31, whereas Richardson et al. (2013) showed resumes of applicants aged 54 to less likely be hired than those of equally qualified applicants aged 42 or 48.

These correspondence audit studies show age discrimination to be one of the reasons why older workers have a higher chance of dropping out of the labor force (Neumark et al., 2016). A survey of the AARP Public Policy Institute also revealed that 51% of older unemployed workers (aged 47–70) reported to be discriminated against because of their older age (Koenig et al., 2015). In support of this, Wanberg et al. (2016) showed negative relationships between job seekers’ chronological age, reemployment status, and reemployment speed. Moreover, these negative relations became stronger over age 50 (see also Perry et al., 2016). Comparable findings have been reported in several other Western countries outside the United States, showing less positive callbacks for older job seekers when compared to their equally qualified younger counterparts (e.g., Krings et al., 2011; Åslund and Skans, 2012), indicating age discrimination in resume screening to be a widespread, substantial, and pressing issue.

### Anonymous Resume Screening

To avert age discrimination in the first phase of the screening procedure, policy makers as well as researchers recommended AAP (see Edin and Lagerström, 2006; Furtmueller et al., 2010; Åslund and Skans, 2012). Furtmueller et al. (2010, p. 10), for instance, concluded that “since employers are prohibited to select employees based on gender, birth date, nationality and marital status, resume forms should not ask for this personal information.” Anonymous resume screening omits explicit demographic cues from resumes that are non-job-related, like date of birth, ethnic-sounding name, or gender (Edin and Lagerström, 2006; Åslund and Skans, 2012; Krause et al., 2012; Hiemstra et al., 2013).

Intriguingly, however, studies have shown that anonymous resume screening may not be as effective as typically assumed as ethnic minority or otherwise stigmatized applicants (e.g., like female or older applicants) are still more rejected when they apply anonymous compared to equally qualified ethnic majority or their non-stigmatized counterparts (Behaghel et al., 2015; Maurer, 2016). For instance, in a field experiment in Germany (Krause et al., 2012), female applicants were less likely to receive a job interview invitation for a *post doc* position in economics compared to equally qualified male applicants when they applied with an AAP. In France, organizations were less likely to invite minority applicants when they received anonymous resumes (Behaghel et al., 2015). The French government, therefore, abandoned the idea of making anonymous resume screening mandatory in public employment service offices. Recently, the Behavioral Economics Team of the Australian Government (BETA) also showed that de-identifying applications for senior positions decreased the number of female and ethnic minority applicants shortlisted for senior positions in the Australian Public Service (Hiscox et al., 2017). Corroborating these findings, a recent scenario study in which both American and European participants had to screen resumes of chronologically older/younger applicants (Kaufmann et al., 2016) revealed that hiring intentions of the chronologically older applicants (i.e., who applied with resumes that included age cues) did not differ significantly from those of the ‘anonymous’ candidates (i.e., who applied with resumes without age cues).

In a Dutch study, Hiemstra et al. (2013) showed that in the absence of demographic information (as in anonymous resume screening), real recruiters still gave lower job suitability ratings to resumes of ethnic minority applicants compared to those of their majority counterparts. Whereas human capital factors could explain these findings to some extent, Hiemstra et al. (2013) could not exclude hiring discrimination, either. Specifically, other resume information (like applicants’ affiliations) than explicit signals of one’s demographic background (like applicants’ chronological age or ethnic background) might operate as ‘implicit’ or subtle markers of applicants’ protected group status and affect hirability ratings (Dovidio and Gaertner, 2000; Cole et al., 2007; Derous et al., 2012).

Indeed, over the past decades, workplace discrimination has become more subtle (Dovidio and Gaertner, 2000; Rosette et al., 2016). In line with this, one could expect recruiters to also turn their attention to more subtle cues in resumes to gain information. Specifically, recruiters may infer applicants’ protected group membership from subtle cues (like one’s affiliations, Dovidio and Gaertner, 2000; Hiemstra et al., 2013), which contrasts with the often discussed view that anonymity prevents recruiters from favoring majority over minority applicants when credentials are equal, at least in the initial stage of the hiring process. The above-mentioned findings raise the question: Is there more into a resume than one’s date of birth (i.e., or any other explicit age cue, like years of work experience) that might disclose one’s chronological age and might instigate age discrimination in hiring, albeit in more subtle ways?

### Implicit Cues in Resumes

#### Job Market Signaling Theory

Recruitment researchers rely on *job market signaling theory* (Spence, 1973, 1974) to explain how actors determine what information is reliable for making job market choices. In its more general and original sense, signaling theory refers to how individuals (i.e., job applicants and recruiters/organizations) with -partly- conflicting interests will communicate and interpret signals/cues of unknown characteristics (i.e., of the organization/job seeker) to obtain the biggest gains, like getting hired or getting the best employees on board. Hence, signaling systems are characterized by information asymmetry between senders and receivers but are at the same time shaped by mutual interests between signalers and receivers (Connelly et al., 2011).

Typically, signaling theory in recruitment research (i.e., job market signaling theory; see Rynes, 1991) is used to explain the cues *job applicants* use to make inferences about the prospective employer/organization (Carter and Highhouse, 2014). Put differently, the recruitment literature considers how observable recruitment characteristics (like recruiter behavior) serve as signals or cues of the unknown (to job seekers) organizational quality or organizational characteristics. A warm recruiter, for instance, may signal to applicants an organization that looks after its employees (Slaughter et al., 2004). However, *recruiters* also look for ‘cues’^2^ about applicants’ overall and unknown qualities in their resumes (Popken, 1993; Cable and Gilovich, 1998; Cole et al., 2003, 2009; Aguinis et al., 2005; Burns et al., 2014). According to the normative-predictive model of resume screening (Vieira Campos Proença and Valente Dias de Oliveira, 2009; Guion, 2011), resume screening should be based on objective and job-related information like applicants’ work experiences and educational background as mentioned in resumes (Cole et al., 2007). Objective and job-related information (like qualifications) might function as ‘explicit cues’ about applicants’ competencies (Bangerter et al., 2012). Yet, Popken (1993) indicated that recruiters also reply upon inference and indirect speech acts when they read resumes. Cole et al. (2003, 2009) indeed showed recruiters to infer applicants’ personality (like agreeableness) from work experiences as mentioned on resumes (see also Burns et al., 2014). Recently, Kaufmann et al. (2016) even showed trait-related inferences from resume pictures to lower applicants’ hiring chances (i.e., with pictures of applicants with old-appearing faces triggering impressions of low health and fitness). Hence, recruiters also tend to infer subjective attributes and even personality characteristics from resume content in an indirect way (from educational credentials, work experiences, and so on), albeit often not in very accurate ways (see Apers and Derous, 2017; Cole et al., 2007). Put differently, some objective and job-related information in resumes might also function as ‘implicit cues’ about other applicant characteristics (like attributes and traits) than this information is originally intended to be used for.

Interestingly, most studies consider qualification-based inferences (e.g., cues to applicants’ personality) but do not consider *social group status inferences* (i.e., cues to applicants’ protected group status, like age), which may also affect recruiters’ impressions of applicants’ overall job qualification (i.e., given age-based inferences) and even impact recruiters’ hiring judgments. For instance, applicant skills as mentioned in resumes might signal applicants’ overall qualification in a very explicit way but at the same time might communicate something else being useful to recruiters, namely applicants’ chronological age (Abrams et al., 2016). Whereas job market signaling theory mainly focuses on actions taken by senders to communicate positive, imperceptible qualities of the signaler to the receiver (like acquired skills), other information could also be conveyed (i.e., co-vary with positive qualities) that might turn-out to be less beneficial or even harmful to senders (like their older age). Implicit cues to one’s social group status, therefore, can be considered as an unintended consequence of actions taken by senders to communicate positive qualities through resume information (Connelly et al., 2011). As shown by Abrams et al. (2016), applicants’ skills’ set (i.e., observable signal of one’s qualifications) might co-vary with receivers’ perceptions of applicants’ chronological age (i.e., indirectly cause chronological age is not explicitly mentioned in the applicants’ skills or any other section). Hence, when job applicants provide explicit information in their resumes about their skills (like ‘being a rapid decision-maker’; ‘understanding others’ views well’), they might *indirectly* signal their age too (when ‘being a rapid decision-maker’ is associated with chronologically younger age and ‘understanding others’ is associated with chronologically older age) which might affect recruiters’ stereotypical impressions of the applicant’s potential productivity. In doing so, recruiters may subtly factor-in job-irrelevant information, like one’s social group status, that they infer from implicit age cues in applicant resumes (Hiemstra et al., 2013; Abrams et al., 2016).

Implicit cues (e.g., regarding one’s chronological age) differ further from explicit cues in that they may be much more ‘hard-to-fake’ by applicants and ‘hard-to-resist’ by recruiters (Bangerter et al., 2012; Abrams et al., 2016). First, cues are considered ‘hard-to-fake’ (or ‘honest’) if signaling happens beyond one’s conscious control regarding one’s unobserved qualities (Connelly et al., 2011; Bangerter et al., 2012). Information about one’s social group status (e.g., age) is implicitly conveyed through ‘honest’ cues, meaning that these cues convey truly useful information to the receiver about the sender’s social group status in an indirect, non-manipulable way (i.e., beyond the sender’s awareness; see Bangerter et al., 2012). Second, implicit cues are ‘hard-to-resist’ by recruiters. Recruiters might look for such implicit, hard-to-fake cues in resumes because applicants are less likely to consciously cheat on these implicit age cues as applicants might not be aware of the age-related associations they indirectly send to recruiters through this information (e.g., skills as ‘proxy’ of applicants’ chronological age; Abrams et al., 2016). Given that applications are characterized by information asymmetry between applicants and recruiters, such information might be of specific interest to recruiters. Moreover, when implicit cues refer to one’s social group status, they may increase category salience and lessen recruiters’ ability to inhibit categorization (Fiske et al., 1999), which may make them even much ‘harder-to-resist.’ Hence, implicit age cues may fuel recruiters’ categorization processes and ageist hiring preferences in rather subtle ways, as discussed next.

#### Implicit Age Cues

The present study integrates predictions from theories that explain hiring discrimination (i.e., cognitive models of impression formation) with job market signaling theory by considering recruiters’ use of age-related cues in resumes. First, according to impression formation theories (Fiske et al., 2002), resumes may trigger social categorization processes and instigate hiring discrimination because of the limited amount of individuating information (i.e., a one or two-page resume). Second, given the limited amount of individuating information on resumes and given that resume-screening is characterized by information asymmetry (Bangerter et al., 2012), recruiters may particularly look for both explicit and implicit cues about applicant characteristics to base their hiring decisions upon. Certain of these cues may signal applicants’ age and may trigger age-related associations (like applicants’ physical and psychological ‘fitness’) to recruiters. Whereas effects of explicit age cues on recruiters’ hiring decisions have already been demonstrated (e.g., using correspondence audit tests; see Neumark et al., 2016), effects of implicit age cues remain largely understudied and are considered here. Specifically, given that implicit cues are ‘hard-to-fake’ and ‘hard-to-resist,’ one could expect strong categorization effects from implicit age cues, thereby affecting job suitability ratings of applicants with old vs. young-age cues in a different way.

Two implicit age cues of interest to this study are applicants’ *first names* and *extracurricular activities*. First, as the popularity of *first names* changes over time (Christopher, 1998; Sigurd et al., 2005), names might indicate in a subtle, indirect way a person’s chronological age and applicants might not be aware of such a subtle and honest signal (i.e., age-related association) in their resumes. Furthermore, because first names are tied with one’s social and personal identity, they may activate social categorization processes and ageism (Young et al., 1993; Bennington and Wein, 2002; King et al., 2006; Derous et al., 2009). Therefore, first names might be both hard-to-fake (applicant perspective) and hard-to-resist (recruiter perspective). For instance, Young et al. (1993) and Christopher (1998) both showed that recruiters infer age from applicants’ first names. Moreover, age associations seemed hard to resist as equally qualified applicants with young-sounding names were perceived more positively and received higher job suitability ratings than those with old-fashioned names, showing there is more into a resume than one’s date of birth (or any other explicit age cue) that might disclose one’s chronological age and instigate age discrimination (Perdue et al., 1990; Rudman et al., 1999).

Second, recruiters also evaluate applicants based on their *extracurricular activities/affiliations with socio-cultural groups* (Dovidio and Gaertner, 2000; Cole et al., 2007; Derous et al., 2009). Cole et al. (2007) showed that if asked directly, recruiters considered extracurricular activities as the least important resume characteristic in judging applicants’ employability. Actual employability ratings, however, showed exactly the opposite, hence indicating that extracurricular activities were factored in (hard-to-resist) when recruiters evaluated applicants. This is buttressed by studies showing ethnic affiliations to lower ethnic minorities’ job suitability ratings (Dovidio and Gaertner, 2000; Derous et al., 2009): Affiliations with certain socio-cultural groups seem to affect recruiters’ information processing and decision-making in subtle ways. Berger (2009) also suggests that extracurricular activities in resumes can be beneficial to older applicants when such activities counter stereotypical inferences about the (older) applicant. Hence, as with names, extracurricular activities might serve as hard-to-fake cues about applicants’ chronological age, particularly when applicants are unaware of the age-related associations and such cues seem hard-to-resist from a recruiter’s perspective (Cole et al., 2007; Abrams et al., 2016).

Building further on both impression formation (Fiske et al., 2002), and job market signaling theory (Bangerter et al., 2012) to explain age discrimination in resume-screening, we expected effects of implicit age cues in such a way that:

**Hypothesis 1.** Applicant resumes with old-sounding names (Hypothesis 1a) and old-fashioned activities (Hypothesis 1b) will receive lower job suitability ratings than those from equally qualified applicants with young implicit age cues.

The traditional view of bias considers membership in a particular social group (e.g., with a protected status) as having the same effect on employment outcomes for all members of that group; typically studies do not consider multiple cues in conjunction and within-category differences (i.e., differences between members from the same social category; see Kaiser and Pratt-Hyatt, 2009; Marcus and Fritzsche, 2014). That is, compared to a single cue on a resume (e.g., name only) one would expect multiple cues (like old-sounding name and old-fashioned extracurricular activities) to increase category salience and lessen the ability of even a motivated decision maker to inhibit the activation of a social category (Kulik et al., 2007; Derous et al., 2017). Similarly, research has suggested that minority group members may be rejected in proportion to their ‘outgroupness’ (Crisp and Hewstone, 1999). Several studies offer evidence that multiple cues to minority membership may lead to greater hiring discrimination (Uhlmann et al., 2002; Segrest Purkiss et al., 2006; Derous et al., 2009, 2015). Segrest Purkiss et al. (2006), for instance, found that two ethnic cues (name; accent) led to more negative interviewer reactions than one cue only. Derous et al. (2009) also showed that the strength of applicants’ ethnic in-group identification (or social category salience) as appearing on resumes affected their job suitability ratings with highly Arab-identified minority applicants receiving lower job suitability ratings compared to equally qualified but less highly Arab-identified applicants. Similarly, because category salience will affect attention to that category (Kulik et al., 2007) and because protected group members (like older workers) may be rejected in proportion to their degree of identification with the protected social group of interest (Kaiser and Pratt-Hyatt, 2009), we expected for implicit age cues that:

**Hypothesis 2.** Applicant resumes with more implicit cues referring to older age will receive lower job suitability ratings than those with less implicit cues referring to older age.

#### Recruiter Age

Recruiters may differ in their susceptibility to bias. Given the limited number of studies that considered effects of recruiter characteristics on ageism in hiring (Kulik et al., 2000) and in resume-screening in particular (Fasbender and Wang, 2017), we focus on recruiters’ chronological age as a potential boundary condition of implicit age effects. Two competing perspectives have been put forward regarding implicit social evaluation. On the one hand, implicit evaluations may favor members from the in-group (i.e., in-group favoritism). In a simulated hiring study, Krings et al. (2011) found recruiters’ own age to attenuate age bias to some extent: The probability of selecting the older candidate instead of the younger candidate increased with increasing age of the evaluator. Specifically, age bias was no longer observed or even turned into older worker favoritism when recruiters were equal in age or older than the older job candidate himself. These findings support the idea of in-group favoritism. Recently, Axt et al. (2014) showed that for both race and gender, the hierarchy of implicit evaluations places in-group members at the top, therefore also evidencing in-group favoritism. Indeed, to preserve one’s social identity, one might favor in-group members (Tajfel and Turner, 1979). Interestingly, however, Axt et al. (2014; see also Nosek et al., 2002) showed a peculiar feature of implicit *age* effects: The hierarchy of implicit evaluations did *not* reflect in-group favoritism for age. Instead, older participants also placed older adults at the bottom of the social hierarchy (Axt et al., 2014). Thus, older adults were preferred considerably less than younger adults, also by older-aged participants, which runs counter to the idea of in-group favoritism as found in the context of implicit race and gender evaluations. Corroborating with this, Christopher (1998) also reported older-age effects (based on first names) among both younger and older participants. Why implicit in-group favoritism does not occur for age cannot be inferred from these data. Yet, Marcus and Sabuncu (2016) recently suggest evolutionary explanations for ageism. Individuals may tend to systematically avoid and even discriminate against older individuals that are reminiscent of potential sickness or decline, in order to protect one’s social group status and one’s individual ego from the threat of sickness, decline, and eventually death. Since thoughts about decline and death tend to particularly operate at the implicit level (Levy and Banaji, 2002), they may exacerbate the negative effects of implicit old-age cues on both older and younger recruiters’ hiring decisions. Hence, evolutionary theories of ageism consider prejudice and discrimination against older applicants as a ‘defense mechanism’ that equally affects older and younger evaluators. In line with these assumptions, one could also expect the opposite, namely a *lack* of in-group favoritism from the part of the older-aged recruiter in case he/she evaluates resumes with implicitly mentioned old-age cues. Yet, given the limited empirical evidence in the context of resume-screening, we formulated the following research question on the potential effect of recruiters’ chronological age:

**Research question.** Will applicant resumes with more implicit age cues referring to older age receive *higher* job suitability ratings from chronologically older recruiters than younger recruiters (*in-group favoritism*) or will applicant resumes with more implicit old-age cues receive equally low job suitability ratings from older recruiters as from younger recruiters (*lack of in-group favoritism*)?

## Materials and Methods

### Ethics Statement

The study was carried out in accordance with the guidelines of the ‘General Ethical Protocol for Scientific Research at the Faculty of Psychology and Educational Sciences’ of the Ethical Commission of the Faculty of Psychology and Educational Sciences at Ghent University, which is the relevant university institutional review board that considers ethical aspects. In accordance with the Declaration of Helsinki, participants provided informed written consent prior to their participation. Participants were debriefed after all the data were collected.

### Participants

Participants of the main study were HR professionals in organizations who regularly recruited applicants and who were identified through researchers’ professional contacts, databases, and networks. In total, 1424 HR professionals were emailed the study link to participate, of which 45.86% (*N* = 653) agreed to participate. Of this group, 93.42% (*N* = 610) were eligible because they recruited applicants on a daily base (i.e., spending about 38% of their daily activities on recruiting). Hence, the final sample comprised 610 participants who recruited applicants frequently (also called ‘recruiters’ or ‘raters’) with a mean age of 41.15 years (*SD* = 11.25 years), 50.70% males, of which 87% had a university degree (bachelor or higher), and about 1/3rd held a lower/junior (32.8%), middle/senior (36.4%), or higher (24.9%) position.

### Procedure and Design

A field-based randomized experimental study (i.e., resume-screening experiment over the internet) was conducted. Participants received an email with an url and personal code that asked for participation in a study on the development of a tool aimed to train/assess recruiters’ competencies (see Derous et al., 2015, for a similar approach). To mask the study purpose to a further extent and to reduce potential item priming, we also included several filler items (Podsakoff et al., 2003).

After having completed the informed consent form, participants read a job description for a ‘project manager’ (i.e., age-neutral as pilot tested; Perry, 1994). Subsequently, participants read and evaluated four resumes (i.e., equally qualified, see ‘Development of study materials and pilot studies’). Specifically, we conducted a 2 (Date of birth: absent vs. present) by [2 (Name: young vs. old-sounding) by 2 (Extracurricular activities: modern vs. old-fashioned)] mixed factorial design. Date of birth (i.e., explicit cue about applicants’ chronological age) was the between-subjects factor, with chronologically younger applicants born in 1987 and older applicants in 1959. The younger applicants were 26 years’ old (born in 1987) whereas the chronologically older applicants were 54 years’ old (born in 1959) at the moment of the data collection. These birth years were selected because previous studies showed more age discrimination for people over 30, and especially when being over 50 (Albert et al., 2011; Ahmed et al., 2012; Richardson et al., 2013). Name and Extracurricular activities (i.e., implicit age cues) were the within-subjects factors. We paired the explicit age cue with the corresponding (young vs. old-sounding) name on the job applicants’ resumes to avoid unrealistic combinations (Christopher, 1998). Job applicants’ sex was kept constant (male applicants only^3^), and applicants’ resumes were counterbalanced to avoid order effects. The dependent variable was job suitability (or hirability rating; see ‘Measures’). After the resume-sifting task, participants completed a filler task, consisting of several distractor items (e.g., asking how they screen applicants). As part of this filler task, we also asked participants to indicate applicants’ age based on names and affiliations (i.e., *manipulation checks*). In the end, participants were asked to fill-out an ‘opinion survey’ that included measures of old-age stereotypes^4^, social desirability, and participants’ demographics. We ended the study with an open-ended probe to ask for any suspicion regarding the study purpose. After data were collected, participants were debriefed.

### Development of Study Materials and Pilot Studies

Prior to the main study, study materials (i.e., advertisement and resumes) were developed and a series of pilot studies (*N*_total_ = 183) were conducted to ensure relevance and equivalence of study materials. (see Supplementary Material for a more detailed description).

First, 25 jobs were selected to evaluate whether these jobs were equally accessible for older and younger workers. Given that there is evidence for the effect of job-related age stereotypes on hiring outcomes (Perry, 1994; Abrams et al., 2016), we aimed to select an age-neutral job. Results of *Pilot Study 1* (*n* = 47, *M*_age_ = 27.39, *SD*_age_ = 5.1, 68% males) showed that the job of ‘project manager’ was perceived as equally accessible for younger and older workers and therefore selected for this study.

Second, implicit age cues were developed and pilot tested. Applicant names were selected based on the annual statistical reports of the local government and onomastics that indicate the popularity of first names in the geographical area where this study was conducted. We also selected 24 extracurricular activities. Based on the results of *Pilot Study 2* (*n* = 60, *M*_age_ = 27.86, *SD*_age_ = 7.51; 73% males), we selected ‘Fons’ and ‘Frans’ as names of older people and ‘Jens’ and ‘Niels’ as names of younger people. We further selected old-fashioned extracurricular activities (i.e., being a member of a bridge club; being a pigeon/finches fancier; being a walking club member) and extracurricular activities that are seen as modern/typically performed by younger people (i.e., being a member of boy scouts; being a snowboarder; being a life board crew member/rescuer).

Third, we listed all other information needed for the resume template (i.e., educational degree/level, work experiences, language, IT proficiency, home address) based on actual resumes posted on job search websites in the area of interest (note that identifying information was deleted). We had to make sure this information to be relevant, age neutral, and equivalent across resumes (as in the case of work experiences) and had to keep this resume information constant (as in the case of language, IT proficiency, home address/neighborhood). Based on *Pilot Study 3* (*n* = 76, *M*_age_ = 25.91, *SD*_age_ = 9.8, 65.4% males), we therefore selected a ‘Master of Science degree in economics’ (relevant as pilot tested) and four work experiences (equivalent as pilot tested). Finally, we held language (English, French, German, and Dutch) and IT proficiency (SAP, MS office, team foundation server) constant as well as applicants’ neighborhood (middle class).

Based on the results of the pilot studies, we then integrated all information to create the final resumes. In sum, the resume template included information about the applicant’s name (old-sounding vs. young-sounding first names as pilot tested), sex (male), date of birth (born in 1987 vs. 1959), home address (middle-class area), relevant educational background and level (Master of Science degree in economics as pilot tested), relevant work experiences (without any indication of the number of years of professional/work experience and considered equivalent across resumes as pilot tested), language and IT proficiency (held constant), and extracurricular activities (modern vs. old-fashioned activities as pilot tested).

### Measures

After participants observed the job ad and the four resumes, they responded to questions using a 5-point Likert-type response scale (unless otherwise mentioned). *Job suitability* was measured with a 4-item measure adapted from Derous et al. (2009). An example item is “Given all information you read about this applicant, how suitable do you believe this applicant is for this function?” (1 = *not suitable at all* to 5 = *very suitable*). Cronbach’s alpha for the four resumes ranged from 0.91 to 0.93 (see **Table 1**). Second, to check *manipulations*, participants rated applicants’ perceived age based on the job applicant’s name and extracurricular activities using the following, self-constructed item: “With what age do you associate [extracurricular activities] or [name of applicant as appearing on the resume]” (1 = *very young* to 5 = *very old*). Third, to control for *social desirable responding*, eight items were adapted from the Impression Management Scale (BIDR, Paulhus, 1991). An example item is: “When I hear people talking privately, I avoid listening” (1 = *strongly disagree* to 5 = *strongly agree*). Cronbach’s alpha was 0.70. Finally, participants indicated *demographics* including their chronological age (*open answer*), their sex (0 = *female*; 1 = *male*), educational level (1 = *college*; 2 = *university*), job level (1 = *lower*, 2 = *middle*, 3 = *higher level*) as well as recruiting experience. Recruiting experience was measured with one self-constructed item, namely “How much time do you spend on recruiting activities on a daily base?” (1 = *1–30*% to 4 = *70–100*%).

**Table 1:** Descriptives, reliabilities and correlations among study variables.

		*M*	*SD*	1	2	3	4	5	6	7	8	9	10
													
(1)	Job suitability ‘Jens’^a^	3.79	0.67	(0.92)									
(2)	Job suitability ‘Frans’^b^	3.54	0.71	0.361ˆ**	(0.91)								
(3)	Job suitability ‘Niels’^c^	3.21	0.76	0.461ˆ**	0.307ˆ**	(0.93)							
(4)	Job suitability ‘Fons’^d^	3.32	0.70	0.310ˆ**	0.328ˆ**	0.395ˆ**	(0.91)						
(5)	Social desirability	3.73	0.55	0.069	–0.006	–0.011	0.010	(0.70)					
(6)	Chronological age^e^	41.15	11.3	0.066	–0.099ˆ*	–0.089ˆ*	–0.111ˆ**	0.114ˆ**	–				
(7)	Recruiting experience^f^	1.37	0.83	–0.031	0.078	0.094ˆ*	0.088ˆ*	–0.015	–0.359ˆ**	–			
(8)	Gender^g^	0.51	0.50	–0.033	–0.084ˆ*	–0.147ˆ**	–0.086ˆ*	–0.085ˆ*	0.333ˆ**	–0.275ˆ**	–		
(9)	Educational level^h^	1.87	0.33	0.058	–0.043	0.021	–0.050	0.031	–0.063	0.063	–0.097ˆ*	–	
(10)	Job level^i^	1.92	0.77	0.008	–0.070	–0.025	–0.042	0.095ˆ*	0.270ˆ**	–0.174ˆ**	0.288ˆ**	0.015	–

*Note. Cronbach’s alpha are on the diagonal. ^a^‘Jens’: Resume with young-sounding name and modern extracurricular activities; ^b^‘Frans’: resume with old-sounding name and modern extracurricular activities; ^c^‘Niels’: resume with young-sounding name and old-fashioned extracurricular activities; ^d^‘Fons’: resume with old-sounding name and old-fashioned extracurricular activities. ^e^Chronological age of the participants. ^f^Only participants who actually recruited applicants were included in the final sample (*N* = 610); recruiting experiences were coded as: 1 = less than or about 30% recruiting activities per day, 2 = 30–50% recruiting activities per day; 3 = 50–70% recruiting activities per day; 4 = 70% or more recruiting activities per day. ^g^Gender: 0 = female; 1 = male. ^h^Educational level: 1 = college, 2 = university. ^i^Job level: 1 = lower, 2 = middle, 3 = higher. ^∗^*p* < 0.05, ^∗∗^*p* < 0.01.*

## Results

### Preliminary Analyses

Before testing the hypotheses, preliminary analyses were conducted to check model assumptions, randomization, manipulations, intercorrelations and potential covariate variables. *First*, we tested assumptions of normality and homogeneity of variances, which were met for each of the applicant resumes. Inspection of the PP-plots, skewness and kurtosis showed that distributions were approximately normal, so that there is support for the assumption of normality. (Since the repeated measure variables only had two levels, assumption of sphericity was not tested.). Levene’s test further showed that homogeneity of variances was met for all repeated measures. *Second*, female and male applicants were equally distributed across conditions, *χ^2^*(1,610) = 0.22, *p* = 0.64, and experimental conditions did not differ from each other in participant age, *F*(1,608) = 0.15, *p* = 0.70, participants’ educational level, *χ^2^*(1,610) = 0.74, *p* = 0.39, job level, *χ^2^*(2,574) = 0.32, *p* = 0.85, and recruiting experience, *χ^2^*(3,610) = 4.19, *p* = 0.24, supporting the assumption of randomization.

*Third*, manipulation checks were successful: Young-sounding names were perceived as significantly younger (*M* = 2.07; *SD* = 0.47) than old-sounding names (*M* = 4.00; *SD* = 0.47), *t*(609) = 59.82, *p* = 0.00. Old-fashioned activities were perceived as significantly older (*M* = 4.09; *SD* = 0.52) than modern activities (*M* = 1.96; *SD* = 0.46), *t*(609) = -64.64, *p* = 0.00.

*Fourth*, inspection of the correlation table (see **Table 1**) indicated that correlations were not overly strong and in line with what could be expected (e.g., no relation between job suitability ratings of any of the applicant profiles on the one hand and social desirable responding, educational level, and job level on the other hand). Finally, as literature suggests that recruiters’ gender and recruiting experience might affect resume evaluations (e.g., Cole et al., 2003, 2007; Waung et al., 2015), we also checked whether participants’ gender and recruiting experience needed to be controlled for in the main analyses (Bernerth and Aguinis, 2016). Gender and resume screening experience related significantly to the job suitability ratings of some of the applicant profiles/resumes. However, because further analyses showed that homogeneity of regressions was not supported, participants’ gender and recruiting experience did not appear to be good covariates and therefore were not included in the final analyses^5^ (Weinfurt, 1995; Tabachnick and Fidell, 2007).

### Testing of Hypotheses and Research Question

**Table 1** presents descriptives, reliabilities, and correlations among study variables. Hypotheses were tested with a series of mixed analyses of covariances and simple effects analyses, given the experimental set-up and nature of this study (see Derous et al., 2015, for a similar approach). Hypothesis 1 investigated whether applicant resumes with old-sounding names (Hypothesis 1a) and old-fashioned activities (Hypothesis 1b) would receive lower job suitability ratings than those from equally qualified applicants with young implicit age cues (i.e., young-sounding names; modern activities) in their resumes. Job suitability was lower for resumes with old-sounding names than young-sounding names, *F*(1,608) = 9.32, *p* < 0.01, η^2^ = 0.02, and old-fashioned activities than modern activities, *F*(1,608) = 278.91, *p* < 0.001, η^2^ = 0.31, thereby supporting effects of implicit age cues (Hypotheses 1a and 1b were supported). Interestingly, results further showed that job suitability was lowest when the explicit age cue (i.e., date of birth) was *omitted* from resumes, *F*(1,608) = 5.32, *p* = 0.02, η^2^ = 0.02 (see **Table 2**).

**Table 2:** Results of mixed analyses of variance for job suitability.

Source	*df*	*F*	*p*	η^2^
Between subjects				
Date of birth (A)	1	5.32	0.02	0.02
Error (A)	608	(0.26)		
Within subjects				
Name (B)	1	9.32	0.00	0.02
Date of birth (A) × Name (B)	1	0.56	0.45	0.00
Error (B)	608	(0.36)		
Activities (C)	1	278.91	0.00	0.31
Date of birth (A) × Activities (C)	1	0.69	0.41	0.00
Error (C)	608	(0.34)		
Name (B) × Activities (C)	1	73.72	0.00	0.11
Date of birth (A) × Name (B) × Activities (C)	1	0.53	0.47	0.00
Error (B × C)	608	(0.27)		

*Note. The between-subjects factor refers to the explicit age cue (date of birth: absent vs. present) whereas the within-subjects factors refer to the implicit age cues (name/extracurricular activities: old vs. young) on resumes. The pattern of results remained the same when participants’ recruiting experience and gender were controlled for.*

**FIGURE 1:**
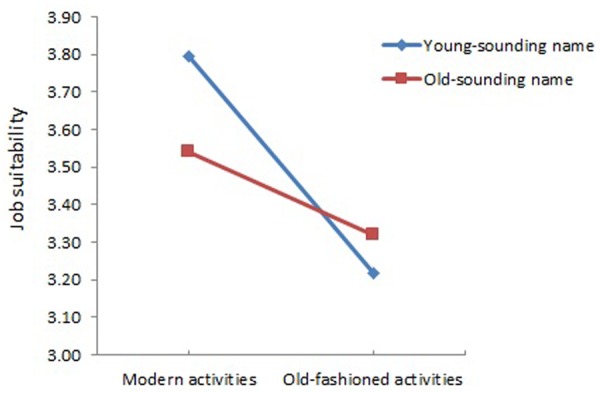
Interaction of implicit age cues (names; extracurricular activities) in resumes on job suitability ratings.

Hypothesis 2 further investigated whether resumes with more implicit cues referring to older age would receive lower job suitability ratings than those with less implicit cues referring to older age. A significant two-way interaction was found for implicit age cues (Name × Extracurricular activities), *F*(1,608) = 73.72, *p* < 0.001, η^2^ = 0.11, and this effect did not depend on explicit age cues, *F*(1,608) = 0.53, *p* = 0.47, η^2^ = 0.00. Simple effects analyses (i.e., to break down the interaction term) further showed significant effects of each of the repeated measures variable (i.e., name or activities) at levels of the other repeated measures variable (i.e., activities or name, respectively) with *p* = 0.00 (in all cases). Inspection of simple effects and **Figure 1** showed that the resume with both a young-sounding name and modern activities received the highest ratings, followed by the resume with an old-sounding name/modern activities, and the resume with an old-sounding name/old-fashioned activities. The resume with an old-sounding name and old-fashioned activities was rated significantly *higher* than the one with a young-sounding name and old-fashioned activities (**Table 2** and **Figure 1**). Hypothesis 2, therefore, was partially supported.

**FIGURE 2:**
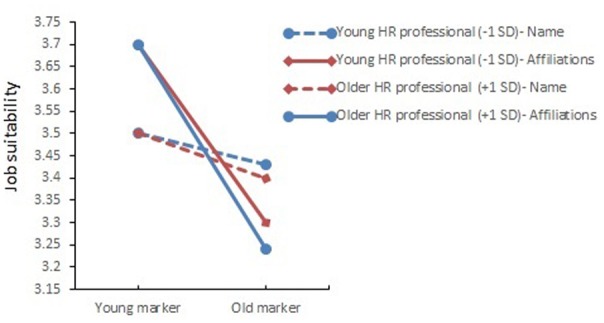
Moderating effects of recruiters’ chronological age and implicit age cues (names; extracurricular activities) on job suitability ratings.

Finally, we formulated a research question to explore potential effects of recruiters’ chronological age. Specifically, we questioned whether resumes with more implicit cues referring to the applicant’s older age would receive higher job suitability ratings when recruiters were chronologically older, or whether applicant resumes with more implicit cues referring to the applicant’s older age would receive equally low job suitability ratings from both older and younger recruiters? Results showed that participants’ chronological age moderated effects of implicit age cues (i.e., name and activities) on applicants’ job suitability ratings, *F*(1,607) = 6.07, *p* = 0.014, η^2^ = 0.01. The test of within-subjects effects further showed that chronologically older participants gave lower scores to resumes of applicants with old-sounding names, whereas no differences were found for resumes of applicants with young-sounding names, *F*(1,607) = 6.73, *p* = 0.01, η^2^ = 0.01. In a similar vein, chronologically older participants gave lower scores to resumes of applicants with old-fashioned activities, whereas no differences were found for resumes of applicants with modern activities, *F*(1,607) = 6.31, *p* = 0.01, η^2^ = 0.01 (see **Figure 2**).

## Discussion

Intrigued by the ongoing debate about the usefulness of anonymous resume screening in many Western societies (e.g., Behaghel et al., 2015; Maurer, 2016), coupled with limited studies that considered AAP effectiveness and subtle mechanisms in hiring discrimination, this study investigated whether omitting explicit age cues (like date of birth) might be beneficial to older applicants or whether more implicit/subtle age cues in resumes may still affect older job applicants’ hirability ratings. Further, because research in personnel recruitment and selection has often failed to consider differences in raters’ tendencies to differentiate among applicants (e.g., Fasbender and Wang, 2017), we investigated raters’ chronological age as a potential moderator.

### Overall Findings

Antidiscrimination legislation and diversity policies typically focus on the impact of explicit age markers on resumes. Unfortunately, the *implicit age cues* on hiring decisions may pass somewhat unnoticed. Building on the principles of job market signaling theory, we found support for the assumption that recruiters make inferences about applicants’ age based on implicit (i.e., ‘hard-to-fake’ and ‘hard-to-resist’) cues in resumes (i.e., applicant first names; extracurricular activities), even in the absence of explicit age cues. Specifically, resumes with both a young-sounding name and modern activities received the highest job suitability ratings, followed by resumes with an old-sounding name/modern activities, and resumes with an old-sounding name/old-fashioned activities. This provides support for so-called ‘within-category’ effects (i.e., Kaiser and Pratt-Hyatt, 2009): Resumes with more implicit cues referring to older age received lower job suitability ratings than less strongly ‘old-age’ identified resumes or those from presumably younger applicants.

Remarkably and somewhat unexpectedly, resumes with a young-sounding name and old-fashioned activities received the lowest job suitability ratings. Because this resume might not have matched the ‘prototypical’ image of young applicants, recruiters might have rated this applicant lowest in overall suitability due to attributional discounting (Kelley, 1973). Indeed, as young applicants are expected to engage rather in modern activities than old-fashioned activities, young applicants (as perceived on the basis of their young-sounding name) who do not do so might have disconfirmed and discounted the dominant young age stereotypes.

Furthermore, omitting *explicit age cues* led to overall lower job suitability ratings. In many Western European societies (as where this study was conducted) it is good practice to indicate date of birth on one’s resume. Not doing so might deviate from the social norm and result in overall lower ratings. Alternatively, implicit age cues might have subtle though stronger effects in the absence of explicit age cues, given that they are hard-to-fake by applicants and hard-to-resist by raters. Moreover, some resume information (like unexplained interruptions in one’s work history) might not be understood properly when recruiters do not know the applicant’s socio-demographic background (like age). Although this was not the case in the present study design (and no significant interaction of the implicit age cues with the explicit age cue was found, either), it has been suggested that anonymous resume screening might not be ideal when there are structural differences between majority-minority applicants (like disparate length of unemployment or educational attainment) as explicit age cues (like age) might also explain, contextualize, and alleviate any subgroup differences (Behaghel et al., 2015). The latter could be investigated to a further extent.

Finally, compared to applicant characteristics, *recruiter characteristics* have been investigated to a considerable lesser extent in hiring discrimination literature (Finkelstein and Burke, 1998); we explored HR professionals’ chronological age to address this gap. First, individuals of different ages may differ in their attitudes toward older adults (Gordon and Arvey, 2004). Indeed, and although effects were small, older participants compared to younger participants gave somewhat lower job suitability scores to older applicants than to younger applicants. Interestingly, this finding does not support in-group favoritism which predicts participants *to prefer* rather than to disfavor in-group members. According to Levy and Banaji (2002), the psychologically permeable nature of boundaries between age groups might allow one to dissociate from his/her own age group. This finding further seems to fit evolutionary theories on (implicit) ageism, that explain bias against older persons as a function of mortality salience, and – at the individual level- as one’s fear of aging and a way to avoid psychologically and physically weak and parasitized individuals to protect one’s own ego and sense of self-esteem (Martens et al., 2005; Marcus and Sabuncu, 2016). Alternatively, findings also remind of the ‘black sheep effect’ (Marques et al., 1988; Brewer, 2007): Older recruiters may reject in-group members as a self-enhancement strategy (i.e., positive distinctiveness) or as a manner of securing one’s position in an organization (i.e., optimal distinctiveness). Finkelstein and Burke (1998) found similar results on in-group bias. However, they used the availability heuristic to explain why older raters disfavored older applicants when applicants’ age was highly salient and when the raters identified with their age groups. Specifically, older HR professionals might be more aware of potential risks when hiring peers (e.g., negative attributes, more expensive, early retirement intentions; Kite et al., 2005; Rau and Adams, 2014). Younger generations of workers, on the other hand, may grow-up in a climate where discrimination issues receive much more attention than previously was the case, which may make younger HR professionals somewhat more cautious about age-related bias.

One might also consider other recruiter characteristics than chronological age, like recruiters’ old-age stereotypes. Old-age stereotypes typically include views that older people are less productive, economically beneficial, competent, creative, flexible, and harder to train (Finkelstein and Burke, 1998; Kulik et al., 2000; Fiske et al., 2002). However, old-age stereotypes are not unequivocal negative in nature (Fiske et al., 2002). Finkelstein et al. (2013), for instance, revealed a balanced view of older workers’ stereotypes, with many positive older-worker stereotypes (e.g., well-mannered, strong work ethic, and reliable). Hence, old-age stereotypes may affect hiring decisions in a complex way (Krings et al., 2011). Yet, few studies considered recruiters’ old-age stereotypes as potential moderators of hiring decisions (Krings et al., 2011; Lu et al., 2011; Fasbender and Wang, 2017). Research might therefore include validated measures of old-age stereotypes (like the Work-related Age-based Stereotypes scale; Marcus et al., 2016). Also more implicit measures of old-age stereotypes might be particularly interesting when one aims to investigate effects of implicit older-age cues on resumes (like the Implicit Association Test; see Levy and Banaji, 2002; Axt et al., 2014).

In sum, this paper explored one recruiter characteristic that might contribute to implicit old-age bias in the context of resume screening, namely recruiters’ own chronological age. No evidence was found for in-group favoritism, which is in line with previous findings on implicit age attitudes (Axt et al., 2014). Older-age bias seems a pressing and complex issue. Yet, as illustrated, and with the exception of a few studies (e.g., Marcus and Sabuncu, 2016; Fasbender and Wang, 2017), its psychological roots are still less well understood, particularly as regards implicit age bias, and therefore in need for further investigation.

### Limitations and Further Research Opportunities

As with any study, limitations need to be mentioned. First, we used a hypothetical job with job applicants in the form of ‘paper’ people (Landy, 2008). Yet, in an early phase of the application procedure, real applicants *are* ‘paper’ people as the only information we have is the information applicants provide on their resume. Moreover, in preliminary screening situations, individuating information about applicants is still limited, therefore, the use of paper people/resumes is ecologically valid in this setting (Copus, 2005).

Second, in our study we employed a mixed-factorial design with implicit age cues as within-subjects factors, which is more realistic than using them in a between-subjects manner (i.e., as this is what recruiters do, namely comparing applicants’ resumes to each other), but which has also been criticized for its potential to inflate hiring discrimination scores (e.g., Bal et al., 2011). A meta-analysis on age bias in evaluations (i.e., hiring, promotion, and performance appraisal) by Gordon and Arvey (2004), however, showed higher mean *d*’s for between-subjects designs than for within-subjects designs. Nevertheless, given that age may become more salient if operationalized in a within-subjects manner, follow-up studies may use between-subjects designs to further investigate implicit age effects.

Third, scenario-based studies can also be criticized on the ground of their lower external validity, but serve other purposes than field studies (like correspondence audit tests) as they may be conducted in a more controlled way and may test contingencies surrounding discriminatory decisions in hiring. Future studies, however, could investigate more and other moderators related to the applicant (like gender; Sidanius and Veniegas, 2000), the job (like type of job, differences in responsibility, and decision-making power; Abrams et al., 2016), and the recruiter (like prejudices and other individual difference variables of relevance; see Self et al., 2015; Fasbender and Wang, 2017). For instance, Brtek and Motowidlo (2002) and Self et al. (2015) showed that recruiters’ accountability might affect discriminatory decision-making, with some types of accountability (like identity-blind accountability) leading to less bias and more objective decision-making than other types of accountability (like identity-conscious accountability). In our scenario-based study, accountability was not primed in any way and participants were randomized over conditions. Therefore, we believe accountability may not have played a large role in affecting findings. Further research on implicit age effects, however, might either control for types of rater accountability or might look at potential effects of recruiters’ accountability on implicit age effects (e.g., by priming accountability). Further, despite our initial attempt to consider both recruiters’ chronological age and older worker stereotypes as potential moderators of implicit age effects, we refrained from further investigating and reporting effects of raters’ older-age stereotypes because of the suboptimal psychometric properties of the ‘Beliefs About Older Workers’ scale (Hassell and Perrewe, 1995) in our sample. Researchers interested in investigating moderating effects of recruiters’ older worker stereotypes may use more recently developed and validated scales such as, for instance, the ‘Beliefs about Older Workers’ Ability and Desire for Learning and Development’ scale (Maurer et al., 2008) or the ‘Work-related Age-based Stereotypes’ scale (Marcus et al., 2016).

Fourth, we distinguish explicit from implicit age cues. Whether cues are rather ‘implicit’ or ‘explicit’ may depend on the nature of the (protected) group status under consideration. For instance, applicants’ names may signal in a rather explicit, direct way one’s ethnic group membership (e.g., Mohammed vs. Mark), but in a rather implicit, indirect manner one’s age. Furthermore, according to signaling theory, implicit age cues are more ‘hard-to-fake’ (or ‘honest’). However, one could argue that both names and extracurricular activities are ‘fakeable’ to some extent. This would be the case when applicants are aware of the particular cues and associations they communicate through affiliations/names (Bangerter et al., 2012).

Another potential study limitation to our field-based, randomized experimental study may be non-response bias. About half of the HR professionals we emailed the study link also participated in this study, which is considerable. Yet, as the likelihood of non-response bias is inversely related to the response rate, this also means that about 50% of respondents did not participate in the study for some or another reason. Non-response bias may be a threat to the external validity of an experimental study if non-respondents’ profiles and answers differ substantially from the profiles and answers of those who did respond to the study (Stone-Romero, 2002). We were not able to log personal information of non-respondents, nor were we able to register reasons of non-response. Given that participants were randomly assigned to the experimental conditions, one might expect non-response also to be random. Yet, whether non-response is random should be investigated empirically. Therefore, further research should take this potential limitation into account, for instance, by finding ways to examine reasons for non-response and by controlling for non-respondents’ demographic attributes.

Finally, this study was conducted in a Western European country. Whereas there seems little evidence of a connection between cultural practices and recruitment and selection practices (see Ryan et al., 2017 for a recent review), cross-cultural differences could exist in the interpretation of age-related cues and attitudes toward older workers (e.g., North and Fiske, 2015), which was not considered here and may warrant further investigation.

### Theoretical and Practical Implications

Our study aimed to extend work on age bias in resume screening in several ways. First, although the Nobel Prize winning paper of Spence (1973) included hiring as an example (Bangerter et al., 2012), relatively little research has applied Spence’s job market signaling theory to (age-based) discrimination in recruitment and more particularly to the act of resume screening. Studies that applied job market signaling theory in recruitment typically considered how applicants interpret unobservable characteristics from signals sent by employers/recruiters through recruitment devices (like job advertisements; see Carter and Highhouse, 2014). Recruitment, however, is a two-way process in which applicants also send signals or cues upon which recruiters/employers may make hiring decisions, which we considered here. Second, research suggests that bias has become more subtle (Rosette et al., 2016), but few studies have measured this in the context of early screening and resume screening in particular, which we did. Hence, with our study, we extend applications of job market signaling theory in recruitment by considering how employers/recruiters may interpret age-related information from subtle cues in job applicants’ resumes. Third, studies that did consider recruiters’ inferences and indirect speech acts mainly focused on applicants’ qualifications including personality inferences (Cole et al., 2007; Aguinis et al., 2005; Burns et al., 2014; Apers and Derous, 2017) but not on social group status, which we investigated and -to the best of our knowledge- has not been considered much. Finally, given the widespread use of resume screening there is a growing interest in studying age effects in this screening stage. Although job applicants’ age shows no validity for predicting future job performance (Schmidt et al., 2016), age discrimination seems substantial as evidenced by a large number of recently conducted correspondence audit studies. Correspondence audits offer a great amount of control and are a very powerful tool to detect labor market discrimination but fail to examine unobserved factors, such as recruiter characteristics. Our study adds to the literature on age discrimination in hiring that just began to investigate contingencies in recruiters (see for another example: Fasbender and Wang, 2017).

Findings might also be relevant to practitioners. When more individuating information about candidates becomes available in later stages of the screening procedure (such as in the job interview or assessment centers), category-based biases might have less of a chance to color decisions; however, this suggests the critical importance of ensuring a lack of discrimination at the earliest stage of resume screening. Anonymous resume sifting may be one tool to level the playing field, but is much debated by HR professionals and society at large (Maurer, 2016; Hiscox et al., 2017). Indeed, anonymous resume screening might be much more complex than it appears at first glance and our results bear that out. First, omitting explicit cues to one’s chronological age (like one’s date of birth) led to overall lower job suitability ratings, and this might have to do with codes of conduct (i.e., what recruiters deem appropriate to be mentioned in resumes). However, it has also been suggested that explicit age cues in resumes might help recruiters understand to a better extent some other, potentially stigmatizing resume information (Behaghel et al., 2015) and -hence- make recruiters even more attentive/sensitive to ageism, which could paradoxically *counter* age-related bias. Finally, blind screening is at odds with targeted recruitment initiatives if one aims to hire for more diversity (Newman and Lyon, 2009).

Whereas names are considered as identifying information and –hence- blotted in anonymous resume screening, extracurricular activities are typically not blotted. Yet, HR professionals still made age inferences based on implicit age cues like extracurricular activities. This raises the question whether one needs to entirely eliminate resumes or whether structured sifting processes with competency and experience checklists should be considered instead of blotting personal information? For instance, some consider resumes to be ‘dead’ and have moved to requiring anonymous work samples from applicants (Feintzeig, 2016), which seems promising given the fact that work samples mirror relevant future work behavior (Joseph, 2016). Despite this promising approach, applicants’ socio-demographic group characteristics (such as age) will always become apparent at later stages of the hiring procedure. Therefore, any effect of (partially) blind recruiting might be nullified if such procedures do not safeguard against hiring bias in later assessment stages, too.

Given these findings, as well as older raters’ slight tendency to in-group bias, organizations may deploy a mix of strategies to minimize ageism in hiring. Aside from screening and training recruiters for diversity (e.g., in how to deal with/interpret explicit and implicit stigmatizing cues in resume information), team-based hiring consisting of a mixed age group of raters (e.g., both older and younger recruiters) might counter age bias in screening too. Furthermore, both initiatives may signal to job seekers that the organization is committed to diversifying the workforce, at least as regards age (Barber, 1998), thereby also affecting the overall organizational image positively.

### Conclusion

For a better understanding and averting hiring discrimination, one needs to move beyond prevalence studies and investigate determinants of hiring discrimination. Results of an experimental study among HR professionals showed hiring discrimination of older applicants based on implicit age cues in resumes and may help understand mixed effects of anonymous resume screening initiatives. Such findings may help organizational decision makers to understand the complexity of fair hiring and the effectiveness of interventions in order to limit age discrimination upon organizational entry.

## Author Contributions

ED designed, coordinated, helped collecting the data, and wrote the study/paper. JD supervised research assistants and collected data.

## Conflict of Interest Statement

The authors declare that the research was conducted in the absence of any commercial or financial relationships that could be construed as a potential conflict of interest.

## Footnotes

1 The correspondence audit technique is an experimental research technique that allows to compare labor market outcomes of applicants who are equally qualified for a job (i.e., identical in all productive characteristics) but who differ in non-job related or non-productive characteristics like -most typically- demographic characteristics (e.g., gender, ethnicity, and age). In correspondence audit studies matched pairs of equivalent resumes are sent to the same employer and callback is registered as the outcome variable in order to investigate whether differential treatment of applicants can be attributed to hiring discrimination.

2 In layman’s terms ‘cues’ and ‘signals’ are used intertwined (as synonyms). In signaling theory, the notions ‘signal’ and ‘cue’ may differ conceptually from each other in that ‘signals’ refer to traits or characteristics of the sender that might change the behavior of the receiver to the benefit of the sender whereas ‘cues’ refer to traits or characteristics of the sender that might benefit the receiver (Connelly et al., 2011). Given the overall lower labor market outcomes of chronologically older compared to younger persons (Wanberg et al., 2016), in the present paper, implicit resume information referring to an applicant’s older age might be captured more by the notion ‘cue’ than ‘signal.’

3 Only male applicants were considered because men participate more in the labor market where this study was conducted than women and because of the need to remove gender as a potentially confounding factor in the design (Sidanius and Veniegas, 2000; OECD, 2015).

4 We administered the ‘Beliefs About Older Workers Scale’ (Hassell and Perrewe, 1995). Because confirmatory factor analysis with maximum likelihood estimation (Lisrel v. 9.2) showed a bad fit for a one-factor model (i.e., meaning that the scale was not unidimensional as suggested by the authors), we did not consider recruiters’ beliefs about older workers in any further analyses (see also Discussion section).

5 Additional analyses in which participants’ gender and recruiting experience were included did not result in different effects than when gender and recruiting experience were not included (see Results section).

## Abbreviations

AAP: anonymous application procedures
