# Supplementary material for: Implicit Age Cues in Resumes: Subtle Effects on Hiring Discrimination
Source: Front Psychol. 2017 Aug 10;8:1321. doi: 10.3389/fpsyg.2017.01321 (PMC5554369; doi:10.3389/fpsyg.2017.01321)
Supplement: Supplementary file 1 [file Table_A1.docx]

**Appendix A**

| Table A1 | |
| --- | --- |
| *Pilot Testing of Study Materials* | |
| 1. | Job ad (Pilot study 1) |
|  | First, the research team selected a pool of 25 jobs to evaluate whether these jobs were equally accessible for older and younger workers. In a *first pilot study*, participants (*n* = 47, *M* _age_ = 27.39, *SD* _age_ = 5.1, 68% males) further evaluated these 25 jobs on perceived accessibility for young versus older workers (1 = *accessible/typical* *for* *younger workers* to 3 = *accessible/typical* *for* *older workers*). Some jobs were perceived as typical for older people (like ‘librarian’) whereas other jobs were perceived as typical for young people (like ‘fitness coach’). The job of ‘project manager’, however, was perceived as equally accessible for younger and older workers (*M* = 2.08, *SD* = .62) and therefore selected for this study. |
| 2. | Names and extracurricular activities (Pilot study 2) |
|  | Next, a second pilot study was conducted in which participants (*n* = 60, *M* _age_ = 27.86, *SD* _age_ = 7.51; 73% males) evaluated implicit age cues that we intended to manipulate, namely first names of applicants and types of extracurricular activities. In the *second pilot study*, participants first evaluated 26 first names of males (1 = *rather young-sounding name/typical name of a younger person* to 3= *rather old-sounding name/typical name of an older person*). Note that applicant sex (i.e., males) was held constant. As last names were not considered susceptible to sex nor age associations, we did not need to pilot test these last names but instead chose last names that are very common in the geographical area in which this study was conducted. Of all 26 first names, ‘Fons’ (*M* = 2.95, *SD* = .29) and ‘Frans’ (*M* = 2.95, *SD* = .30) outstand as names of older people, whereas the names ‘Jens’ (*M* = 1.23, *SD* = .42) and ‘Niels’ (*M* = 1.39, *SD* = .49) were perceived as names of younger persons. On average, age perceptions of ‘Fons’ and ‘Frans’ (*M* = 1.31, *SD* = .40) differed significantly from average age perceptions of ‘Jens’ and ‘Niels’ (*M* = 2.95, *SD* = .28), *t* (56) = -27.48, *p* < .001. (Note that a series of paired-wise t-tests resulted into the same conclusion when single names were compared to each other, like Jens to Fons). Hence, we selected ‘Fons’ and ‘Frans’ as ‘old-sounding names’ and ‘Jens’ and ‘Niels’ as the young-sounding first names for the resumes.  Next, participants evaluated 24 extracurricular activities on the degree these extracurricular activities are seen as typically being performed by younger versus older people ranging from 1 = *rather* *typical for* *younger people* to 3 = *rather typical for* *older people)*. We chose ‘old-fashioned activities’ and ‘modern activities’ that differed significantly in perceived age. On average, modern activities (i.e., being a member of boy scouts; being a snowboarder; being a life board crew member/rescuer) were perceived as rather typical for younger people (*M* = 1.19, *SD* = .22), whereas the old-fashioned activities (i.e., being a member of a bridge club; being a pigeon/finches fancier; being a walking club member) we selected were perceived as rather typical for older people (*M* = 2.83; *SD* = .33), *t* (56) = 32.35, *p* < .001. (Note that a series of paired-wise t-tests resulted into the same conclusion when single activities were compared to each other, like pigeon fancier to member of boy scouts). |
| 3. | Other resume information (Pilot study 3) |
|  | Finally, other resume information was preselected by the research assistants and its relevance was discussed based on actual resumes. In a *third pilot study*, participants (*n* = 76, *M* _age_ = 25.91, *SD* _age_ = 9.8, 65.4% males) first evaluated information regarding applicants’ educational background/level. A ‘Master of Science degree in economics’ was perceived as a relevant for project manager (*M* = 2.31, *SD* = .55, with 1 = *rather not relevan*t to 3 = *rather* *very relevant*). Second, we tested four relevant work experiences on their perceived equivalence, as preselected and discussed by research assistants (i.e., on age neutrality) and based on actual resumes of project managers. A repeated measures anova showed the four work experiences to be equivalent, *F*(3, 58) = 1.22 *p* = .31. Further, language (English, French, German, and Dutch) and IT proficiency (SAP, MS office, team foundation server) were held constant across resumes as well as the neighborhoods (middle class) in which the applicants lived in order not to induce extra bias. Note that none of the qualifications and work experiences mentioned in the resumes were dated (i.e., without any indication of the number of years of professional/work experience). |
